# Polymorphisms and expression pattern of circular RNA circ-ITCH contributes to the carcinogenesis of hepatocellular carcinoma

**DOI:** 10.18632/oncotarget.18327

**Published:** 2017-06-01

**Authors:** Wenzhi Guo, Jiakai Zhang, Dongyu Zhang, Shengli Cao, Gongquan Li, Shuijun Zhang, Zhihui Wang, Peihao Wen, Han Yang, Xiaoyi Shi, Jie Pan, Hua Ye

**Affiliations:** ^1^ Department of Hepatic and Biliary Pancreatic Surgery, Henan Key Laboratory of Digestive Organ Transplantation, The First Affiliated Hospital of Zhengzhou University, Zhengzhou, Henan, P.R. China; ^2^ Department of Infectious Disease, The First Affiliated Hospital of Zhengzhou University, Zhengzhou, Henan, P.R. China; ^3^ College of Public Health, Zhengzhou University, Zhengzhou, Henan, P.R. China

**Keywords:** circ-ITCH, hepatocellular carcinoma, susceptibility, survival

## Abstract

Hepatocellular carcinoma (HCC) ranks the sixth most common cancer and the third cause of cancer-related mortality worldwide. Recent studies identified that circ-ITCH Suppresses mutiple cancers proliferation via inhibiting the Wnt/beta-Catenin pathway. In current study, conducted a genetic association study together with epidemiological follow-up study to delineate the role of circ-ITCH in the development and progression of HCC. we found rs10485505 (adjusted OR =1.18; 95% CI=1.06-1.31; P value =3.1×10^-3^) and rs4911154 (adjusted OR =1.27; 95% CI=1.14-1.43; P value =3.7×10^-5^) were significantly associated with increased HCC risk. The expression level of circ-ITCH was significantly lower in HCC tissues, compared with that in adjacent tissues (P value < 0.001). Cox regression analysis indicated that high expression of circ-ITCH was associated with favorable survival of HCC (HR=0.45; 95% CI=0.29-0.68; P value < 0.001). These results indicate that circ-ITCH may have an inhibitory effect on HCC, and could serve as susceptibility and prognostic biomarkers for HCC patients.

## INTRODUCTION

Hepatocellular carcinoma (HCC) ranks the sixth most common cancer and the third cause of cancer-related mortality worldwide [1, 2]. In Asia and sub-Saharan Africa areas, over 5% of the populations suffered chronically infected chronic hepatitis B virus (HBV) infection, which could reflect the elevated prevalence of HCC [1, 3–5]. In china, the estimated new cases and deaths for HCC were 466.1 and 422.1 thousands, respectively [6]. While the data for the estimated new cases and deaths for HCC in USA was 39,230 and 27,170, respectively [7]. Although many potential risk factors have been explored for their associations with susceptibility of HCC, the etiological factors and pathogenesis mechanisms underlying HCC development appear to be complex and heterogeneous [3, 8–13].

Circular RNAs (circRNAs) are an enigmatic class of RNA with unknown function [14]. With the development of next-generation sequencing (NGS), especially RNA sequencing technology, over 30,000 circRNAs have already been detected [15]. Emerging studies indicates that circRNAs, serving as potential diagnostic and predictive biomarkers, play important roles in the development and progression of multiple cancers [16–21]. It's reported that circRNAs play a vital role in many aspects of malignant phenotypes, including cell cycle, apoptosis, vascularization, and invasion, and metastasis [15, 22, 23]. Further exploration of the functions of circRNAs as molecular markers in carcinogenesis of cancer will provide potential application perspectives, such as early tumor diagnosis, therapeutic target screening, prognosis prediction, and target-therapy for tumors.

Recently, Li et al [24] found that circ-ITCH had inhibitory effect on esophageal squamous cell carcinoma (ESCC) by suppressing the Wnt/β-catenin pathway. Furthermore, Wan et al [25] replicated the similar mechanism in lung cancer. However, there are no reported studies on the functional roles of circ-ITCH in HCC. In this study, we hypothesized that circ-ITCH might be involved in the carcinogenesis of HCC. SNPs in the circ-ITCH gene region may conclusively affect individual variation in inhibitory effect of circ-ITCH, and modulate individual cancer susceptibility. So investigation of SNPs in circ-ITCH gene contributes to uncovering pathogenesis of HCC. To address this hypothesis, we conducted this genetic association study together with epidemiological follow-up study to delineate the role of circ-ITCH in the development and progression of HCC.

## RESULTS

### Characteristics of study subjects

A totally of 1,600 HCC cases and 1,800 cancer-free controls were recruited for the genetic association study. Table 1 presents the basic characteristics of the studied population. The distributions of most variables, including age, gender, HBV infection status, smoking status, and education level, were comparable between the HCC cases and cancer-free controls (P value > 0.05). However, a significant difference for the distributions of alcohol status and family history of cancer were detected (P value < 0.001).

**Table 1:** Demographic and epidemiological characteristics of the study population

Variables	HCC (N=1800)		Controls (N=1800)		P value
	N	%	N	%	
Gerder					
Male	1170	65.0	1215	67.5	0.113
Female	630	35.0	585	32.5	
Age					
≥55	1102	61.2	1089	60.5	0.657
<55	698	38.8	711	39.5	
HBV infection					
HBsAg +	450	25.0	414	23.0	0.160
HBsAg -	1350	75.0	1386	77.0	
Smoking status					
Ever	369	20.5	360	20.0	0.709
Never	1431	79.5	1440	80.0	
Alcohol status					
Ever	540	30.0	396	22.0	P<0.001
Never	1260	70.0	1404	78.0	
Family history of cancer					
Yes	99	5.5	18	1.0	P<0.001
No	1701	94.5	1782	99.0	
Education level					
<College degree	1458	81.0	1431	79.5	0.258
≥College degree	342	19.0	369	20.5	

### Association analysis between the tagSNPs in circ-ITCH and HCC risk

**Figure 1:**
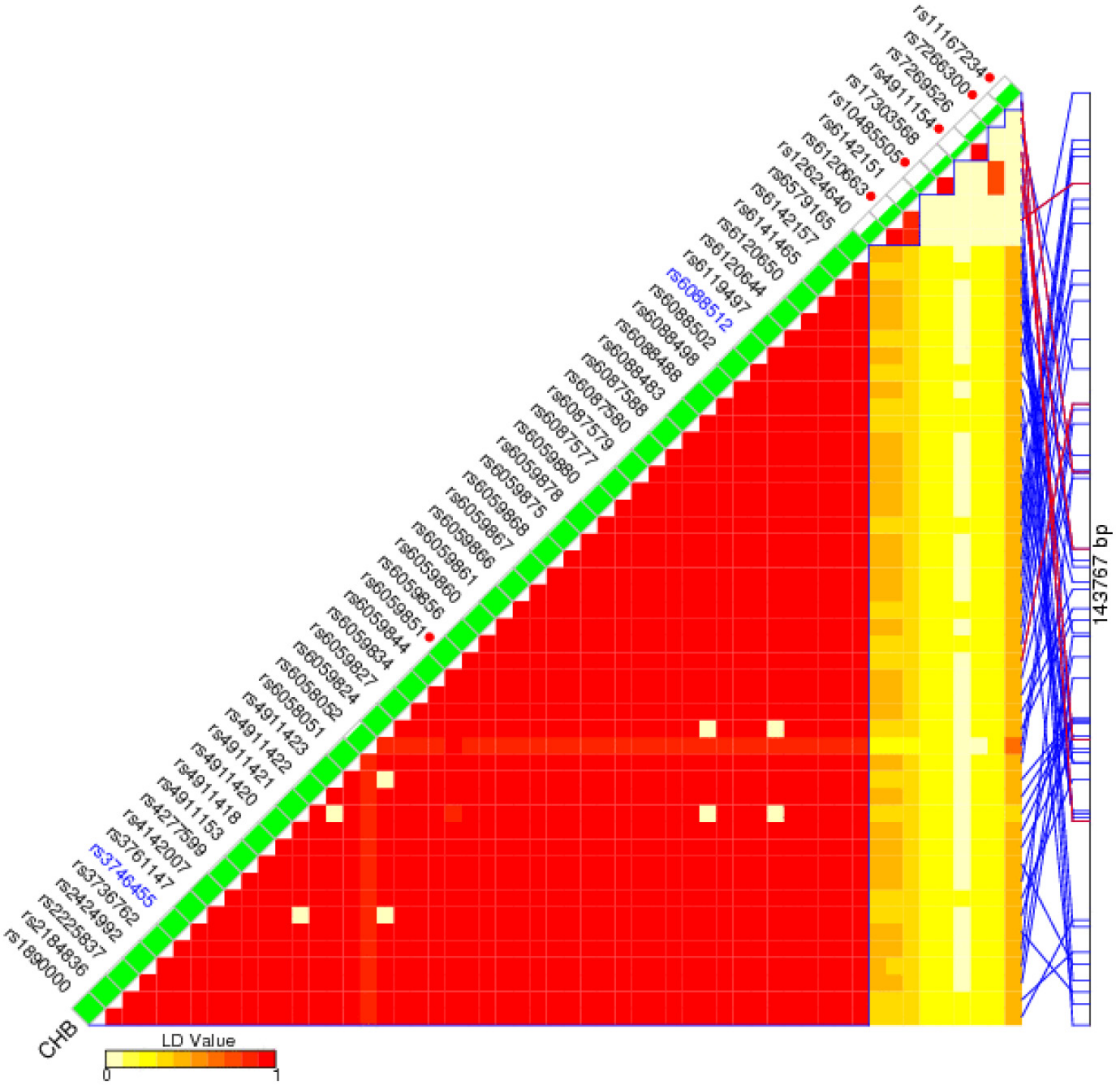
TagSNP selection of circ-ITCH SNPinfo Web Server (https://snpinfo.niehs.nih.gov/) was used for the tagSNP selection of circ-ITCH.

Figure 1 presents the linkage disequilibrium (LD) information of the SNPs located in the gene region of circ-ITCH, during which six SNPs with minor allele frequency (MAF) ≥0.05 in Chinese Han population were selected as tagSNPs. As shown in Table 2, we present the genotype distribution of the selected SNPs and their associations with HCC risk. The genotype frequencies of all six SNPs among both cases and controls were consistent with Hardy-Weinberg equilibrium (HWE) (P value > 0.05). Results of additive model indicated that the genotype distribution of rs10485505 (adjusted OR =1.18; 95% CI=1.06-1.31; P value =3.1×10^-3^) and rs4911154 (adjusted OR =1.27; 95% CI=1.14-1.43; P value =3.7×10^-5^) were significantly associated with HCC risk. For rs10485505, compared with the genotype CC, genotype CT (adjusted OR =1.18; 95% CI=1.03-1.35) and TT (adjusted OR =1.40; 95% CI= 1.04-1.88) were significantly associated with increased HCC risk; while for rs4911154, compared with the genotype GG, genotype GA (adjusted OR =1.27; 95% CI= 1.10-1.46) and AA (adjusted OR =1.74; 95% CI= 1.21-2.49) were significantly associated with increased HCC risk.

**Table 2:** Circ-ITCH genotypes and the risk of HCC

	Cases (N=1800)	Controls (N=1800)	OR (95% CIs) *	P trend
rs6059851				
GG	640 (35.6%)	600 (33.3%)	1.00 (reference)	
GA	878 (48.8%)	870 (48.3%)	1.04 (0.90-1.20)	
AA	342 (19.0%)	330 (18.4%)	1.07 (0.89-1.29)	
A vs G			1.04 (0.94-1.24)	0.452
rs6120663				
CC	1027 (57.1%)	1035 (57.5%)	1.00 (reference)	
AC	685 (38.1%)	673 (37.4%)	1.02 (0.89-1.18)	
AA	88 (4.9%)	92 (5.1%)	0.96 (0.71-1.31)	
A vs C			1.01 (0.90-1.12)	0.912
**rs10485505**				
CC	1001 (55.6%)	1083 (60.2%)	1.00 (reference)	
CT	689 (38.3%)	632 (35.1%)	1.18 (1.03-1.35)	
TT	110 (6.1%)	85 (4.7%)	1.40 (1.04-1.88)	
T vs C			1.18 (1.06-1.31)	3.1×10^-3^
**rs4911154**				
GG	1089 (60.5%)	1200 (66.7%)	1.00 (reference)	
GA	632 (35.1%)	550 (30.5%)	1.27 (1.10-1.46)	
AA	79 (4.4%)	50 (2.8%)	1.74 (1.21-2.49)	
A vs G			1.27 (1.14-1.43)	3.7×10^-5^
rs7266300				
AA	1053 (58.5%)	1002 (55.7%)	1.00 (reference)	
AT	665 (36.9%)	699 (38.8%)	0.90 (0.79-1.04)	
TT	82 (4.6%)	99 (5.5%)	0.79 (0.58-1.07)	
T vs A			0.90 (0.81-1.00)	0.061
rs11167234				
TT	819 (45.5%)	820 (45.6%)	1.00 (reference)	
TA	783 (43.5%)	775 (43.0%)	1.01 (0.88-1.16)	
AA	198 (11.0%)	205 (11.4%)	0.97 (0.78-1.20)	
A vs T			0.99 (0.90-1.09)	0.880

* Adjusted for age, gender, HBV infection, family history of cancer, smoking status, alcohol status, and education level.

### Expression of circ-ITCH in HCC and the adjacent tissues

**Figure 2:**
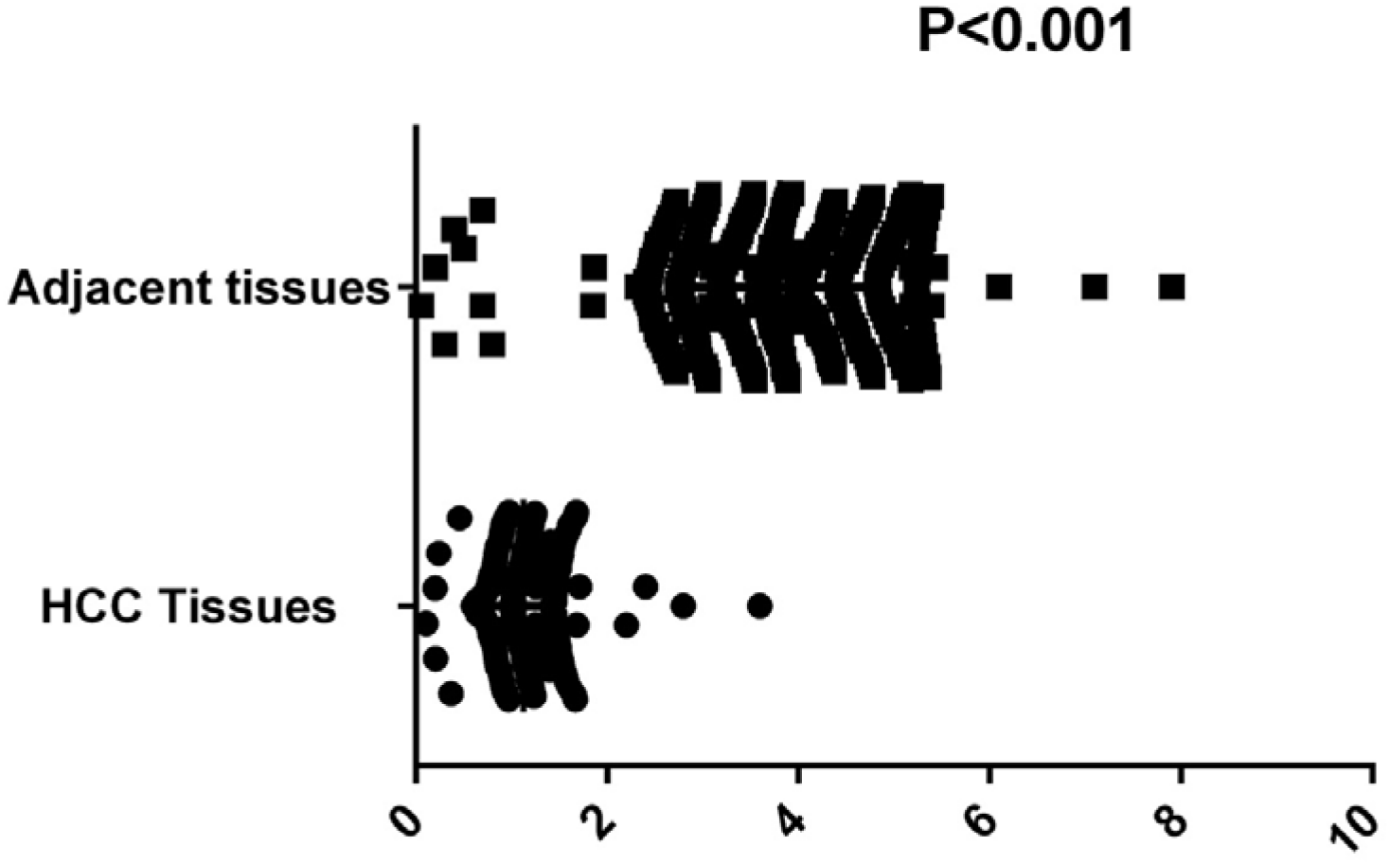
Comparison of circ-ITCH expression levels between HCC tissues and adjacent tissues Mann–Whitney U-test was used to analyze the expression difference of circ-ITCH between HCC tissues and adjacent non-cancerous tissues.

To explore the influence of expression of circ-ITCH on occurrence of HCC, a TaqMan-based qRT-PCR assay was used for the divergent primer set to determine the levels of ITCH in 288 HCC samples and matched adjacent tissues. As shown in Figure 2, the expression level of circ-ITCH was significantly lower in HCC tissues, compared with that in adjacent tissues (P value < 0.001).

### Survival analysis of circ-ITCH in HCC

**Figure 3:**
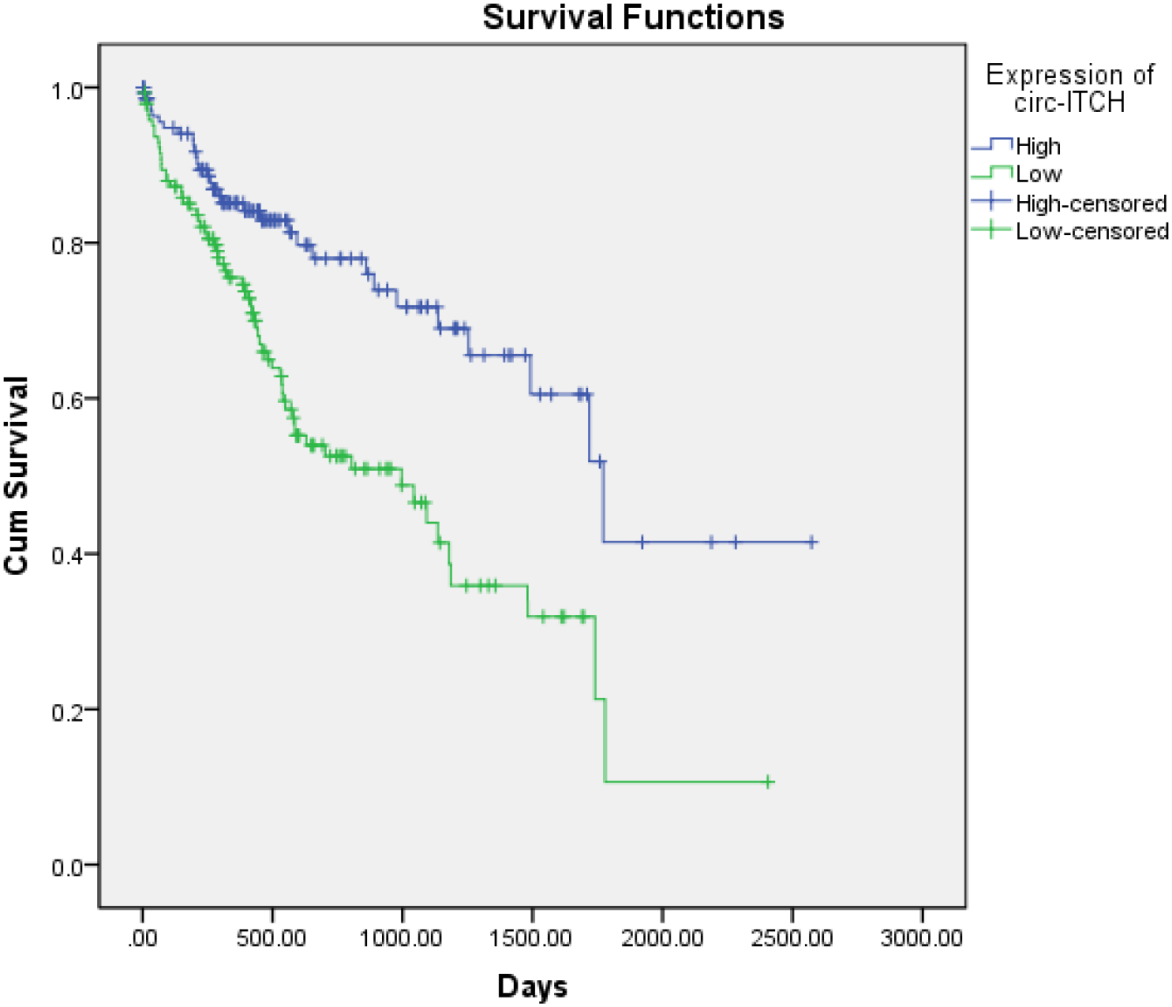
Prognostic value of circ-ITCH Kaplan–Meier curves were utilized to assess the association between the circ-ITCH and overall survival of HCC patients.

Furthermore, to determine whether circ-ITCH could serve as prognostic marker for HCC, we analyzed the association between expression of circ-ITCH and prognosis of HCC in the 288 HCC cases with follow-up data. As shown in Figure 3, the difference between the survival curves of the two groups was statistically significant (P value < 0.001). Cox regression analysis indicated that high expression of circ-ITCH was associated with favorable survival of HCC (HR=0.45; 95% CI=0.29-0.68; P value < 0.001). This indicated that circ-ITCH could work as an prognostic indicator for HCC.

## DISCUSSION

In this study, we have used a candidate gene approach to examine the associations between genetic variations of circ-ITCH and susceptibility of HCC. We also evaluated the possibility of the expression of circ-ITCH as an susceptibility and prognostic biomarker for HCC. To the best of our knowledge, This should be the first study which aims to investigate the role of circ-ITCH in the development and progression of HCC. Our findings demonstrated that rs10485505 and rs4911154 were significantly associated with increased risk of HCC, and the expression of circ-ITCH could serve as susceptibility and prognostic biomarkers for HCC patients.

CircRNAs are a novel type of RNAs, which are generated primarily through a type of alternative RNA splicing called “back-splicing,” a downstream splice donor being joined to an upstream splice acceptor through splice skipping or direct splice [16]. They could be used as molecular markers or potential targets for early tumor diagnosis, therapeutic evaluation, prognosis prediction, and even gene therapy for tumors [15]. Li et al [26] found circRNA could be a critical factor in HCC using RNA-seq data of 100 samples consisting of tumor tissues and para-carcinoma tissues from 50 HCC patients were downloaded from the Sequence Reads Archive (SRP068976). Recent studies demonstrated that circ-ITCH Suppresses mutiple cancers proliferation via inhibiting the Wnt/beta-Catenin pathway [24, 25, 27]. Wnt/beta-Catenin pathway, which regulates some of the crucial aspects of cellular processes, plays important role in the carcinogenesis progress of many cancers [28–38]. ITCH could hyper expression promotes ubiquitination and degradation of phosphorylated Dvl2, thereby inhibiting the Wnt/β-catenin pathway [24]. Song et al [37] reported that Wnt/beta-catenin was also an oncogenic pathway targeted by H. pylori in gastric carcinogenesis. Genetic polymorphisms of Wnt/beta-catenin pathway genes are also associated with the susceptibility, survival, efficacy and toxicities of radiotherapy of multiple cancers [39–45]. In current study, we found the expression level of circ-ITCH in HCC tissues was significantly lower than the level in adjacent tissues, which indicated that circ-ITCH worked as an tumor suppressor gene for carcinogenesis of HCC, consistent with its function in ESCC, colorectal cancer, and lung cancer.

Circ-ITCH was located on chromosome 20q11.22, and spans exons 6–13 of gene ITCH, which encodes a member of the Nedd4 family of HECT domain E3 ubiquitin ligases [46]. Mutations in this gene are a cause of syndromic multisystem autoimmune disease [47, 48]. In the genetic association study, we first found that rs10485505 and rs4911154 in circ-ITCH were significantly associated with increased risk of HCC with a relatively large sample size in a well-established population. Using RegulomeDB [49], the score for rs10485505 and rs4911154 were both ‘1f’, which means Likely to affect binding and linked to expression of a gene target. These data open new perspectives on a possible genetic determinants of HCC in candidate CircRNAs.

Our study has several strengths. First, the large sample size provides enough statistical power to detect such moderate associations. Second, we have included the genetic-homogenous participants, which were all Han Chinese population. Although our study revealed some interesting findings, there still exist some limitations. One limitation was that the findings were not validated in an independent cohort. Secondly, further mechanism of circ-ITCH and its SNP affecting susceptibility and prognosis of HCC was not explored here [50]. However, these new findings still provide a detailed genomic landscape in which to examine biological mechanisms of HCC.

In summary, our study showed that the expression level of circ-ITCH in HCC tissues was significantly lower than the level in adjacent tissues, and high expression of circ-ITCH was associated with favorable survival of HCC. We also found that rs10485505 and rs4911154 in circ-ITCH were significantly associated with increased risk of HCC. This study indicates that circ-ITCH not only has prognostic significance, but also susceptibility biomarker for HCC. Meanwhile, genetic variation of circ-ITCH can also be used as the biomarkers for prediction and screening of HCC. These findings should be verified by larger, well-designed epidemiologic and functional studies.

## MATERIALS AND METHODS

### Subjects

Totally included in current study were 1,600 HCC cases and 1,800 cancer-free controls. All cases were pathologically confirmed as HCC, and all subjects in this study were homogenous Han Chinese. The control subjects were randomly enrolled at the same period when they visit the hospital for physical examinations, matched by age, gender, HBV infection status, and education level. Demographic data and environmental exposure history of each participants were collected by face to face interviews. Blood (10ml) was collected for each patient. Plasma was separated immediately and stored at -80°C before use, while tissue samples were snap-frozen in liquid nitrogen immediately after dissection and then stored at −80°C before RNA extraction. The study was approved by the institutional review board of the hospital, and a written informed consent was collected from all individual participants of the study.

### RNA extraction and real-time quantitative polymerase chain reaction

Total RNA was extracted from 288 randomly selected HCC tissue samples and matched adjacent tissues using TRIzol reagent (Invitrogen, Carlsbad, CA, USA) according to the manufacturer's protocol. The quality of the RNA samples were measured using Agilent 2100 bioanalyzer, and an Integrity Number value above 6.0 indicated acceptable RNA integrity for RT-PCR assays. The relative gene expression of circ-ITCH (primers: the forward GCAGAGGCCAACACTGGAA, the reverse TCCTTGAAGCTGACTACGCTGAG) was determined using the ABI Prism 7900 sequence detection system (Applied Biosystems, Foster City, CA, USA), and GAPDH was used as an internal standard control. All reactions were performed in triplicate.

### SNP selection and genotyping

SNPinfo Web Server (https://snpinfo.niehs.nih.gov/) was used for the tagSNP selection of circ-ITCH. As shown in Figure 1, Six SNPs with minor allele frequency (MAF) ≥0.05 in Chinese Han population, including rs6059851, rs6120663, rs10485505, rs4911154, rs7266300, rs11167234, were selected for this study. Genomic DNA was extracted from peripheral blood of each study subject. TaqMan allelic discrimination assay was used to genotype the selected SNPs by using the ABI 7900HT Real-Time PCR System (Applied Biosystems, Foster City, CA, USA). For quality control, we randomly selected 10% of the samples for repeated genotyping, and the results were 100% concordant.

### Statistical analyses

Student's t test and Pearson's chi-squared (χ2) test were used to assess the differences in demographic factors between HCC cases and controls. Hardy-Weinberg equilibrium (HWE) of the controls was done by a goodness-of-fit χ2 test. The ORs and 95% CIs were calculated by an unconditional logistic regression model for the associations between genotype distribution and HCC susceptibility. Mann–Whitney U-test was used to analyze the expression difference of circ-ITCH between HCC tissues and adjacent non-cancerous tissues. Patients were divided into low or high expression groups of circ-ITCH according to the median value. Kaplan–Meier and Cox regression analyses were utilized to assess the association between the circ-ITCH and overall survival of HCC patients. Statistical analyses were performed by using SPSS software package (Version 13.0, SPSS Inc., Chicago, IL, USA), and all reported p values were two-sided with a significance level of 0.05.

## References

[1] Torre LA, Bray F, Siegel RL, Ferlay J, Lortet-Tieulent J, Jemal A (2015). Global cancer statistics, 2012. CA Cancer J Clin.

[2] Frenette C (2016). Surveillance for hepatocellular carcinoma. Clin Adv Hematol Oncol.

[3] Chen X, Wu F, Liu Y, Lou J, Zhu B, Zou L, Chen W, Gong J, Wang Y, Zhong R (2016). The contribution of serum hepatitis B virus load in the carcinogenesis and prognosis of hepatocellular carcinoma: evidence from two meta-analyses. Oncotarget.

[4] Joung JG, Ha SY, Bae JS, Nam JY, Gwak GY, Lee HO, Son DS, Park CK, Park WY (2017). Nonlinear tumor evolution from dysplastic nodules to hepatocellular carcinoma. Oncotarget.

[5] Li W, Li M, Liao D, Lu X, Gu X, Zhang Q, Zhang Z, Li H (2016). Carboxyl-terminal truncated HBx contributes to invasion and metastasis via deregulating metastasis suppressors in hepatocellular carcinoma. Oncotarget.

[6] Chen W, Zheng R, Baade PD, Zhang S, Zeng H, Bray F, Jemal A, Yu XQ, He J (2016). Cancer statistics in China, 2015. CA Cancer J Clin.

[7] Siegel RL, Miller KD, Jemal A (2016). Cancer statistics, 2016. CA Cancer J Clin.

[8] Chen JS, Liang LL, Xu HX, Chen F, Shen SL, Chen W, Chen LZ, Su Q, Zhang LJ, Bi J, Zeng WT, Li W, Ma N (2016). miR-338-3p inhibits epithelial-mesenchymal transition and metastasis in hepatocellular carcinoma cells. Oncotarget.

[9] Dai L, Peng XX, Tan EM, Zhang JY (2016). Tumor-associated antigen CAPERalpha and microvessel density in hepatocellular carcinoma. Oncotarget.

[10] Han SY, Han HB, Tian XY, Sun H, Xue D, Zhao C, Jiang ST, He XR, Zheng WX, Wang J, Pang LN, Li XH, Li PP (2016). MicroRNA-33a-3p suppresses cell migration and invasion by directly targeting PBX3 in human hepatocellular carcinoma. Oncotarget.

[11] Ji J, Xu M, Zhao Z, Tu J, Gao J, Lu C, Song J, Chen W, Chen M, Fan X, Cheng X, Lan X, Li J (2016). SMAD7 loci contribute to risk of hepatocellular carcinoma and clinicopathologic development among Chinese Han population. Oncotarget.

[12] Jianyong L, Jinjing Z, Jingcheng H, Zhengni L, Peng Z, Lixue W, Lunan Y, Jinqiang Z, Yong Z, Bo L, Tianfu W, Wentao W (2016). Hepatocellular carcinoma cases with high levels of c-Raf-1 expression may benefit from postoperative adjuvant sorafenib after hepatic resection even with high risk of recurrence. Oncotarget.

[13] Kaibori M, Sakai K, Ishizaki M, Matsushima H, De Velasco MA, Matsui K, Iida H, Kitade H, Kwon AH, Nagano H, Wada H, Haji S, Tsukamoto T (2016). Increased FGF19 copy number is frequently detected in hepatocellular carcinoma with a complete response after sorafenib treatment. Oncotarget.

[14] Memczak S, Jens M, Elefsinioti A, Torti F, Krueger J, Rybak A, Maier L, Mackowiak SD, Gregersen LH, Munschauer M, Loewer A, Ziebold U, Landthaler M (2013). Circular RNAs are a large class of animal RNAs with regulatory potency. Nature.

[15] Wang Y, Mo Y, Gong Z, Yang X, Yang M, Zhang S, Xiong F, Xiang B, Zhou M, Liao Q, Zhang W, Li X, Li Y (2017). Circular RNAs in human cancer. Mol Cancer.

[16] Hou LD, Zhang J (2017). Circular RNAs: an emerging type of RNA in cancer. Int J Immunopathol Pharmacol.

[17] Yu L, Gong X, Sun L, Zhou Q, Lu B, Zhu L (2016). The circular RNA Cdr1as act as an oncogene in hepatocellular carcinoma through targeting miR-7 expression. PLoS One.

[18] Yang P, Qiu Z, Jiang Y, Dong L, Yang W, Gu C, Li G, Zhu Y (2016). Silencing of cZNF292 circular RNA suppresses human glioma tube formation via the Wnt/beta-catenin signaling pathway. Oncotarget.

[19] Xuan L, Qu L, Zhou H, Wang P, Yu H, Wu T, Wang X, Li Q, Tian L, Liu M, Sun Y (2016). Circular RNA: a novel biomarker for progressive laryngeal cancer. Am J Transl Res.

[20] Xin Z, Ma Q, Ren S, Wang G, Li F (2017). The understanding of circular RNAs as special triggers in carcinogenesis. Brief Funct Genomics.

[21] Sand M, Bechara FG, Sand D, Gambichler T, Hahn SA, Bromba M, Stockfleth E, Hessam S (2016). Circular RNA expression in basal cell carcinoma. Epigenomics.

[22] Zhu J, Ye J, Zhang L, Xia L, Hu H, Jiang H, Wan Z, Sheng F, Ma Y, Li W, Qian J, Luo C (2017). Differential expression of circular RNAs in glioblastoma multiforme and its correlation with prognosis. Transl Oncol.

[23] Zhang Y, Li J, Yu J, Liu H, Shen Z, Ye G, Mou T, Qi X, Li G (2017). Circular RNAs signature predicts the early recurrence of stage III gastric cancer after radical surgery. Oncotarget.

[24] Li F, Zhang L, Li W, Deng J, Zheng J, An M, Lu J, Zhou Y (2015). Circular RNA ITCH has inhibitory effect on ESCC by suppressing the Wnt/beta-catenin pathway. Oncotarget.

[25] Wan L, Zhang L, Fan K, Cheng ZX, Sun QC, Wang JJ (2016). Circular RNA-ITCH suppresses lung cancer proliferation via inhibiting the Wnt/beta-catenin pathway. Biomed Res Int.

[26] Li Y, Dong Y, Huang Z, Kuang Q, Wu Y, Li M (2017). Computational identifying and characterizing circular RNAs and their associated genes in hepatocellular carcinoma. PLoS One.

[27] Huang G, Zhu H, Shi Y, Wu W, Cai H, Chen X (2015). cir-ITCH plays an inhibitory role in colorectal cancer by regulating the Wnt/beta-catenin pathway. PLoS One.

[28] Adamo A, Fiore D, De Martino F, Roscigno G, Affinito A, Donnarumma E, Puoti I, Ricci-Vitiani L, Pallini R, Quintavalle C, Condorelli G (2017). RYK promotes the stemness of glioblastoma cells via the WNT/ beta-catenin pathway. Oncotarget.

[29] Cho YH, Cha PH, Kaduwal S, Park JC, Lee SK, Yoon JS, Shin W, Kim H, Ro EJ, Koo KH, Park KS, Han G, Choi KY (2016). KY1022, a small molecule destabilizing Ras via targeting the Wnt/beta-catenin pathway, inhibits development of metastatic colorectal cancer. Oncotarget.

[30] Cui Y, Zhang F, Zhu C, Geng L, Tian T, Liu H (2017). Upregulated lncRNA SNHG1 contributes to progression of non-small cell lung cancer through inhibition of miR-101-3p and activation of Wnt/beta-catenin signaling pathway. Oncotarget.

[31] Gao H, Sun B, Fu H, Chi X, Wang F, Qi X, Hu J, Shao S (2016). PDIA6 promotes the proliferation of HeLa cells through activating the Wnt/beta-catenin signaling pathway. Oncotarget.

[32] Ge C, Wu S, Wang W, Liu Z, Zhang J, Wang Z, Li R, Zhang Z, Li Z, Dong S, Wang Y, Xue Y, Yang J (2015). miR-942 promotes cancer stem cell-like traits in esophageal squamous cell carcinoma through activation of Wnt/beta-catenin signalling pathway. Oncotarget.

[33] Guo YH, Wang LQ, Li B, Xu H, Yang JH, Zheng LS, Yu P, Zhou AD, Zhang Y, Xie SJ, Liang ZR, Zhang CM, Zhou H (2016). Wnt/beta-catenin pathway transactivates microRNA-150 that promotes EMT of colorectal cancer cells by suppressing CREB signaling. Oncotarget.

[34] Kong LY, Xue M, Zhang QC, Su CF (2017). *In vivo* and *in vitro* effects of microRNA-27a on proliferation, migration and invasion of breast cancer cells through targeting of SFRP1 gene via Wnt/beta-catenin signaling pathway. Oncotarget.

[35] Kovacs D, Migliano E, Muscardin L, Silipo V, Catricala C, Picardo M, Bellei B (2016). The role of Wnt/beta-catenin signaling pathway in melanoma epithelial-to-mesenchymal-like switching: evidences from patients-derived cell lines. Oncotarget.

[36] Lei JJ, Peng RJ, Kuang BH, Yuan ZY, Qin T, Liu WS, Guo YM, Han HQ, Lian YF, Deng CC, Zhang HJ, Chen LZ, Feng QS (2015). NOP14 suppresses breast cancer progression by inhibiting NRIP1/Wnt/beta-catenin pathway. Oncotarget.

[37] Song X, Xin N, Wang W, Zhao C (2015). Wnt/beta-catenin, an oncogenic pathway targeted by H. pylori in gastric carcinogenesis. Oncotarget.

[38] Wu G, Liu A, Zhu J, Lei F, Wu S, Zhang X, Ye L, Cao L, He S (2015). MiR-1207 overexpression promotes cancer stem cell-like traits in ovarian cancer by activating the Wnt/beta-catenin signaling pathway. Oncotarget.

[39] Yu J, Huang Y, Liu L, Wang J, Yin J, Huang L, Chen S, Li J, Yuan H, Yang G, Liu W, Wang H, Pei Q (2016). Genetic polymorphisms of Wnt/beta-catenin pathway genes are associated with the efficacy and toxicities of radiotherapy in patients with nasopharyngeal carcinoma. Oncotarget.

[40] Kim SS, Cho HJ, Lee HY, Park JH, Noh CK, Shin SJ, Lee KM, Yoo BM, Lee KJ, Cho SW, Cheong JY (2016). Genetic polymorphisms in the Wnt/beta-catenin pathway genes as predictors of tumor development and survival in patients with hepatitis B virus-associated hepatocellular carcinoma. Clin Biochem.

[41] Yilmaz M, Donmez G, Kacan T, Sari I, Akgul Babacan N, Sari M, Kilickap S (2015). Significant association between polymorphisms of Wnt antagonist genes and lung cancer. J Investig Med.

[42] Mostowska A, Pawlik P, Sajdak S, Markowska J, Pawalowska M, Lianeri M, Jagodzinski PP (2014). An analysis of polymorphisms within the Wnt signaling pathway in relation to ovarian cancer risk in a Polish population. Mol Diagn Ther.

[43] Alanazi MS, Parine NR, Shaik JP, Alabdulkarim HA, Ajaj SA, Khan Z (2013). Association of single nucleotide polymorphisms in Wnt signaling pathway genes with breast cancer in Saudi patients. PLoS One.

[44] Gabrovska PN, Smith RA, Haupt LM, Griffiths LR (2011). Investigation of two Wnt signalling pathway single nucleotide polymorphisms in a breast cancer-affected Australian population. Twin Res Hum Genet.

[45] Fernandez-Rozadilla C, de Castro L, Clofent J, Brea-Fernandez A, Bessa X, Abuli A, Andreu M, Jover R, Xicola R, Llor X, Castells A, Castellvi-Bel S, Carracedo A (2010). Single nucleotide polymorphisms in the Wnt and BMP pathways and colorectal cancer risk in a Spanish cohort. PLoS One.

[46] Perry WL, Hustad CM, Swing DA, O'Sullivan TN, Jenkins NA, Copeland NG (1998). The itchy locus encodes a novel ubiquitin protein ligase that is disrupted in a18H mice. Nat Genet.

[47] Shembade N, Harhaj NS, Parvatiyar K, Copeland NG, Jenkins NA, Matesic LE, Harhaj EW (2008). The E3 ligase Itch negatively regulates inflammatory signaling pathways by controlling the function of the ubiquitin-editing enzyme A20. Nat Immunol.

[48] Matesic LE, Haines DC, Copeland NG, Jenkins NA (2006). Itch genetically interacts with Notch1 in a mouse autoimmune disease model. Hum Mol Genet.

[49] Boyle AP, Hong EL, Hariharan M, Cheng Y, Schaub MA, Kasowski M, Karczewski KJ, Park J, Hitz BC, Weng S, Cherry JM, Snyder M (2012). Annotation of functional variation in personal genomes using RegulomeDB. Genome Res.

[50] Zheng J, Deng J, Xiao M, Yang L, Zhang L, You Y, Hu M, Li N, Wu H, Li W, Lu J, Zhou Y (2013). A sequence polymorphism in miR-608 predicts recurrence after radiotherapy for nasopharyngeal carcinoma. Cancer Res.

